# Case of Chronic Peritonitis Developed by Pregnancy

**Published:** 1857-03

**Authors:** Daniel B. Cliffe

**Affiliations:** Franklin, Tennessee


ART. IX.—*A Case of Chronic Peritonitis developed by Pregnancy.* By DANIEL B. CLIFFE, M.D., of Franklin, Tennessee.

DECEMBER 30, 1855, I was called to see Lara, female slave, aged 26, and found her laboring under the following symptoms: muscular system attenuated and flabby; countenance anxious; skin cool and dry; respiration hurried; with frequent expectoration of bloody mucus. The abdomen was quite large, and fluctuation distinct; complained of slight tenderness over the entire abdominal surface on pressure, and of occasional lancinating pains.

She confidently stated that she was five or six months advanced in pregnancy, though no uterine tumor could be distinguished through the abdominal walls.

The tongue was red, smooth, and pointed; appetite bad, and digestion impaired; daily amount of salivary secretion from one to two pints; frequent vomiting of pale green fluid; occasional diarrhœa alternated with obstinate constipation; secretion from the kidneys scanty and high-colored; pulse, 140, small and feeble.

Upon a careful examination with the stethoscope, neither the sounds of the fœtal heart nor the *bruit de souffle* could be heard.

The os uteri could be felt high up, enlarged and slightly patulous, and the uterus itself seemed immovable. She was the mother of several children, and had suffered in previous gestations with symptoms similar to those now present.

With a view of bringing about a more healthy secretion from the various affected organs, she was ordered to take a pill composed of blue mass and ext. tarax., morning and evening; and occasionally through the day a mixture of the solut. bicarb. pot. and tinct. digital. Rubefacients, anodyne fomentations, and vesications, applied over the abdomen.

Under this treatment, her symptoms were somewhat improved; her appetite increased, and digestion became more natural. The salivation and expectoration ceased. The rapidity of the pulse subsided, and hopes were entertained of a favorable issue.

January 14th. She was now ordered iod. pot. and chalybeates. For the succeeding few weeks no appreciable difference presented, except a slight increase in the size of the abdomen, until February 28, when she was seized with labor pains, and, before assistance could reach her, was delivered of a feeble and emaciated infant of about the seventh month, with the membranes entire. Slight flooding ensued, and the uterus contracted as well as in ordinary cases. The abdomen was but slightly diminished in size, and had lost none of its hardness.

Thirty-six hours after the birth of the child, and while at stool, she suddenly expired.

Autopsy 16 hours after death. Upon making a crucial incision through the walls of the abdomen, 3 or 4 pints of limpid yellow serum escaped; and in attempting to raise the flaps, the entire contents of the cavity were found to be firmly adherent to each other as well as to the parietes. It was with no little difficulty that the liver, omentum, and intestines could be separated; strong bands of false membrane uniting them. The uterus was well contracted, although the peritoneal coat was covered with false membrane in every stage of development. The entire surface of the intestines and the serous lining of the abdomen were thickly studded with white opaque bodies, varying from a line to half an inch in diameter, lying between the serous coat and the adventitious membrane.

The bladder was healthy; kidneys not examined; lungs healthy.

The principal point of interest in the foregoing is the previous existence of peritonitis, and recovery with, perhaps, slight adhesions.

These would doubtless have remained harmless for an indefinite period, but for the inopportune occurrence of pregnancy; the advance of which would necessarily interfere with the relative position of the various abdominal viscera and break up any adhesions, thereby exciting inflammation anew. Subacute peritonitis existed most certainly in the pregnancy previous to the last, and probably in one or more previous ones.

